# Old World Cutaneous Leishmaniasis and Refugee Crises in the Middle East and North Africa

**DOI:** 10.1371/journal.pntd.0004545

**Published:** 2016-05-26

**Authors:** Rebecca Du, Peter J. Hotez, Waleed S. Al-Salem, Alvaro Acosta-Serrano

**Affiliations:** 1 Sabin Vaccine Institute and Texas Children’s Hospital Center for Vaccine Development, Departments of Pediatrics and Molecular Virology and Microbiology, National School of Tropical Medicine, Baylor College of Medicine, Houston, Texas, United States of America; 2 Department of Biology, Baylor University, Waco, Texas, United States of America; 3 James A. Baker III Institute, Rice University, Houston, Texas, United States of America; 4 Department of Parasitology, Liverpool School of Tropical Medicine, Liverpool, England, United Kingdom; 5 Department of Vector Biology, Liverpool School of Tropical Medicine, Liverpool, England, United Kingdom; Pasteur Institute of Iran, ISLAMIC REPUBLIC OF IRAN

The Syrian refugee crisis has precipitated a catastrophic outbreak of Old World cutaneous leishmaniasis now affecting hundreds of thousands of people living in refugee camps or trapped in conflict zones. A similar situation may also be unfolding in eastern Libya and Yemen.

Leishmaniasis has been endemic in Syria for over two centuries, with the first case ever reported being as early as 1745, when it was known as the “Aleppo boil” [1,2]. Old World cutaneous leishmaniasis (CL) is characterized most notably by disfiguring skin lesions, nodules, or papules, and in the Middle East and North Africa (MENA) region it is primarily caused either by *Leishmania tropica* (anthroponotic) or *L*. *major* (zoonotic), with some sporadic cases also caused by *L*. *infantum* (Box 1) [3–5]. In North Africa, a chronic form of CL also can be caused by *L*. *killicki* [6–7].

**Box 1. Old World Cutaneous Leishmaniasis (CL) in the MENA Region**

Anthroponotic CL

Major etiologic agent: *Leishmania tropica* [4,5,7]

Major vector: *Phlebotomus sergenti* [4,5]

Zoonotic CL

Major etiologic agent: *L*. *major* [4,5,7]

Minor etiologic agent: *L*. *infantum* [4,5]

Vectors: *Ph*. *papatasi* for *L*. *major*; *Ph*. *perfiliewi*, *Ph*. *perniciosus*, *Ph*. *longicuspis*, and *Ph*. *ariasi* for *L*. *infantum* [5]

Major animal reservoirs: Rodents (*L*. *major*) and dogs (*L*. *infantum*) [4,7]

Although Old World CL is generally not fatal, clinical symptoms can lead to disfiguring scars that result in social stigmatization and psychological consequences. The World Health Organization (WHO) has estimated that around 2.4 million disability-adjusted life years (DALYs) are lost due to CL and visceral leishmaniasis (VL) globally [8]; however, the number of DALYs attributed to CL is still under evaluation. The 2013 Global Burden of Disease Study determined that CL causes only 41,700 DALYs [9], while other studies have found that these figures may represent profound underestimates [10,11].

Studies observing the impact of marring CL facial scars have found that the social stigmatization involved leads to anxiety, depression, and decreased quality of life for patients [12]. The scars can lead to a changed perception of self and can limit individuals’ abilities to participate in society, further decreasing their social, psychological, and economic well-being, as employment opportunities become scarce. Women, adolescents, and children are particularly susceptible to the social stigmatization of disfiguring scars [13]. The hardships caused by CL extend beyond physical symptoms and manifest most prominently in patients’ social, psychological, and economic well-being. Like many neglected tropical diseases (NTDs), CL not only occurs in settings of poverty but the disease also has the ability to perpetuate and reinforce poverty, catalyzing a positive feedback loop between disease and poverty [14]. For many of these reasons, the WHO classifies leishmaniasis as one of 17 NTDs [15], although the cutaneous form is often not prioritized in major global health initiatives, unlike the NTDs now targeted by integrated preventive chemotherapy [11].

## Pre-Conflict Old World CL in Syria

**Fig 1:**
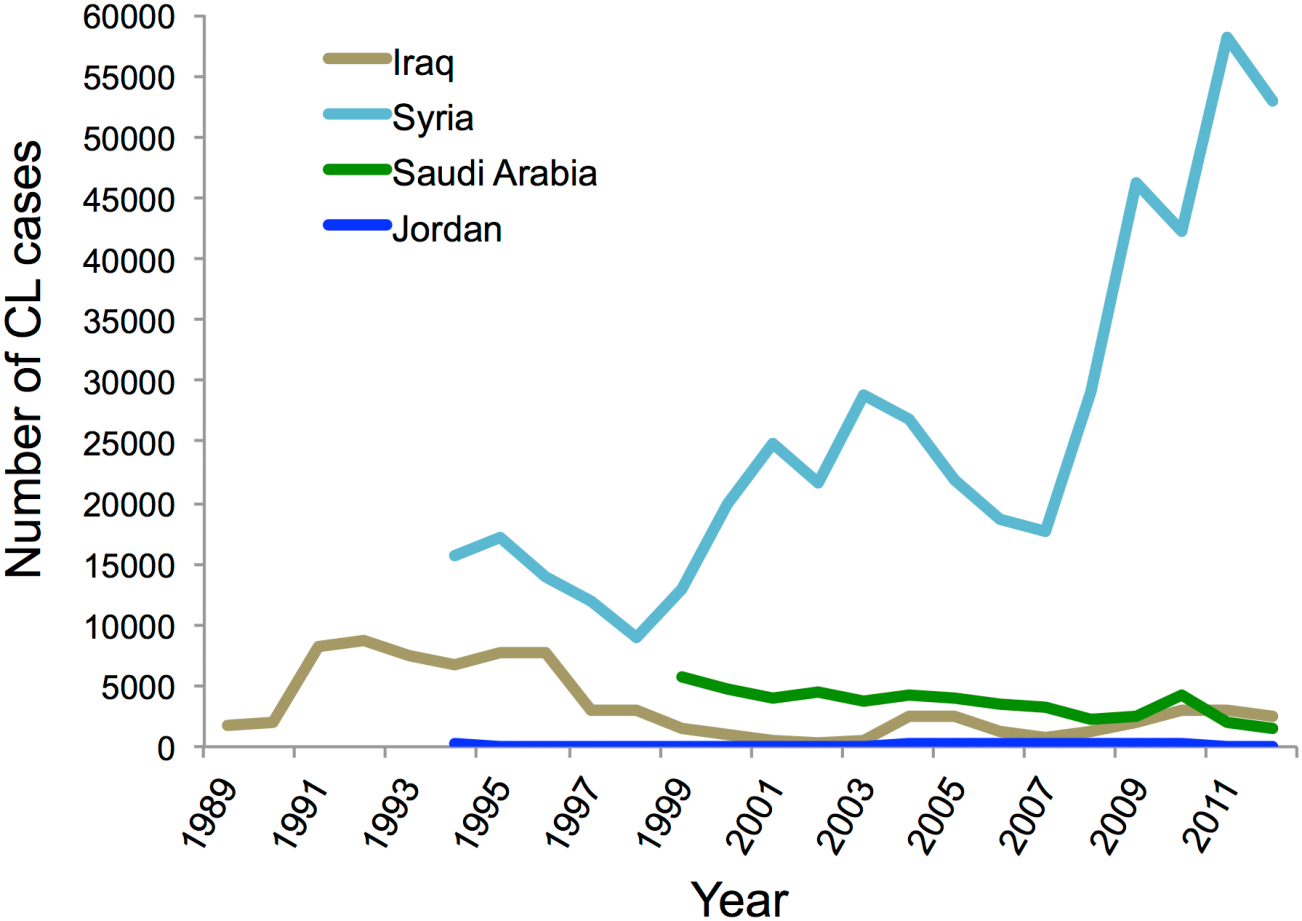
Year-wise trend of CL cases reported in Middle East (from Salam et al 2014, *PLOS Neglected Tropical Diseases*) [20].

Even before the current crisis, the Syrian government has struggled to contain endemic CL. After a 30-year hiatus during which CL was mostly restricted to Aleppo and Damascus [16], CL re-emerged in northwestern Syria in 1988 [1,17]. In 1991, the incidence of CL dropped temporarily due to insecticide spraying, but it began to rapidly rise again even as insecticide spraying continued [18]. The increased number of cases may have been accounted for in part by increased awareness and reporting of the disease; however, the most likely explanation for the dramatic increase and distribution of CL starting in the early 1990s stems from socioeconomic and environmental factors [1]. During this time, Syria experienced rapid and decentralized urbanization as city suburbs expanded and the population density increased [19]. People began to migrate from rural to urban areas, and municipal departments, overwhelmed by these changes, were no longer able to provide adequate hygiene and sanitation services such as trash collection and disposal, as well as insecticide spraying [1,19]. As populations migrated, individuals with no immunity became exposed to CL and the disease spread [1]. Such factors may account for a steep rise in the apparent number of CL cases in Syria beginning in 2008 as reported previously by Salam et al. in *PLOS Neglected Tropical Diseases* (Fig 1) [20].

The numbers of CL cases in Syria began to increase even further following the onset of the Syrian Civil War in March, 2011, with new cases appearing in regions previously thought to be non-endemic.

### War, Refugees, and the Emergence of Catastrophic NTDs

While CL is by no means new to Syria [7], the war in Syria has greatly increased the risk for CL and reports have indicated sharp increases in the number of CL cases in Syria and in surrounding areas of the Middle East [2]. Armed conflict enables outbreaks of serious NTDs [4,14,21,22] due to a combination of factors—most notably, collapsed health care infrastructures and population displacement. As populations migrate to endemic and non-endemic regions, they are exposed to infections for the first time or introduce diseases into new areas, respectively [4,20,23]. Additionally, the chaos and instability often lends to poor living conditions, which further exacerbate the risk for rapid transmission of infectious diseases [4,14]. In recent years, such factors were notable for producing catastrophic NTD outbreaks of cholera in the Democratic Republic of Congo and kala-azar in Sudan [24,25]. Additionally, human migration can be accompanied by deforestation or tumultuous urbanization, which often exacerbate disease outbreaks [26]. In West Africa, all of these factors combined to create a “perfect storm” for the 2014–2015 Ebola virus infection epidemic [27]. The ongoing Syrian conflict has escalated similar factors of instability and chaos that have been shown by past events, such as the Ebola epidemic in West Africa, to facilitate infectious disease outbreaks.

**Fig 2:**
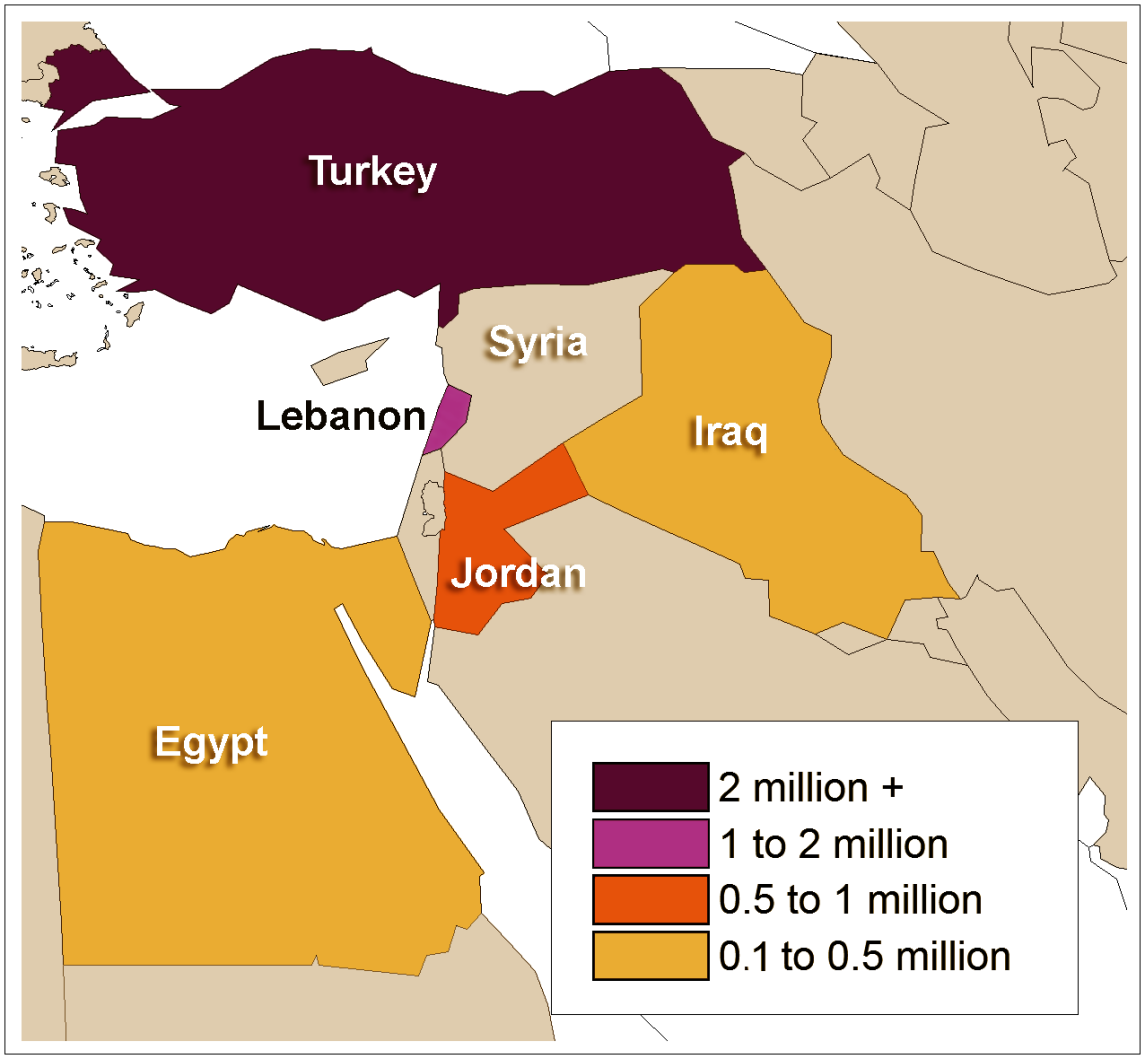
The displacement of Syrian refugees (data from UNHCR, Syria Regional Refugee Response: Inter-agency Information Sharing Portal) [31].

As the conflict in Syria approaches its fifth year, over 50% of the public hospitals in Syria have been destroyed and the health care infrastructure is bordering on nonexistent [28]. Thus far, an estimated 6.5 million Syrians have been internally displaced [29,30] and an additional 4.4 million Syrians have been externally displaced [31]. Due to the violence, Syrians have been forced to flee from their homes and seek refuge across the Middle East, North Africa, and, more recently, Europe [31]; currently, 95% of the over 4 million refugees who have fled Syria reside in Turkey, Jordan, Lebanon, Iraq, and Egypt (Fig 2) [31]. The mass migration of people within Syria and the MENA region has put a strain on resources. Internally displaced individuals have reported that they are in need of non-food items (including personal hygiene products), health care services, food, shelter, water, and education [32]. Similarly, externally displaced individuals are often living in overcrowded and unhygienic spaces, commonly without access to many necessary resources, including basic sanitation and waste disposal services, food, electricity, as well as health care [33–36]; in Lebanon, refugee camps consist mostly of makeshift houses built out of scrap and rubble or tents [33]. Syria is now the leading producer of refugees in the world and has contributed significantly to what is considered to be the largest global refugee crisis since World War II [37,38].

Both inside and outside of Syria, conditions of poverty and malnutrition are prevalent and living situations are grim as adequate municipal services and necessary resources remain scarce [33]. The socioeconomic and environmental circumstances created by the conflict in Syria facilitate risk factors for the continued transmission of CL, and they not only potentiate increased incidence of CL but also exacerbate the morbidity and mortality of CL after transmission [14,33]. Ongoing violence in Syria has corresponded with infrastructural instability and chaotic population migration. It has created a setting in which we have seen the re-emergence of polio and measles, as well as tuberculosis, hepatitis A, and other infections in Syria and among displaced Syrian refugees [30,39]. However, the pathognomonic and obvious clinical features of Old World CL caused by both *L*. *tropica* and *L*. *major* possibly make it the most visible sign of disease emerging under the current circumstances.

## Old World CL and the Current Syrian Crisis

With settings enabling transmission of Old World CL as a backdrop, the number of new cases has continued to rise. Within Syria, a 2013 study published by the Ministry of Health reported an incidence rate more than twice as high as the incidence rate reported in Syria between 2004 and 2008 by the WHO. The annual incidence of CL in Syria between 2004 and 2008 was estimated to be 23,000 cases per year [40]. In 2012, 53,000 cases were reported, and in the first half of 2013 alone, 41,000 cases were reported [41]. Additionally, the number of cases of CL has most likely been severely underreported [42]; the WHO estimated that the actual incidence of CL in Syria between 2004 and 2008 was three to five times higher than the reported incidence [40]. The true number of annual incident and prevalent cases in Syria may therefore exceed 100,000.

Along its border with Syria, Turkey—which has taken more Syrian refugees than any country thus far [31]—has shown indications of increased prevalence of CL among already endemic existing populations in correlation with the influx of refugees from Syria [23]. Old World CL has emerged in Lebanon as well, although the outbreak to date has been largely contained to refugee populations [33,43,44]. A new report indicates that among the cases of CL observed in refugee communities in Lebanon, 85% of the cases are caused by *L*. *tropica*, with the remainder caused by *L*. *major* [33]. This may complicate treatment in the long term as *L*. *tropica* patients tend to be more refractory to the main CL drug, sodium stibogluconate (SSG) [45–47]. In the countries that have observed new cases of CL, younger age groups, due to their lack of previous exposure to the disease, have been the most affected [23,33]. Non-immune existing individuals also are at great risk of contracting CL as their immune systems are not equipped to fend off the parasite.

Few countries have mandated reporting of CL [33], and the resultant weak reporting system promotes a lack of disease awareness and public policies for treatment and prevention. Compounding this problem is the absence of rapid diagnostics and the requirement to have highly skilled dermatologists and pathologists establish a diagnosis on the basis of clinical presentation and confirmatory microscopy, respectively. Even then, the sensitivity of microscopy is not particularly high (68% for *L*. *major* and 45% for *L*. *tropica*) [48]. The CL clinical presentation is also often accompanied by a wide spectrum of clinical manifestations that can mimic other inflammatory and neoplastic diseases, further complicating the diagnosis and reporting of CL [49,50].

If Old World CL is not addressed promptly, experience warns of a likely outbreak that may have unanticipated consequences if allowed to erupt. In the early 2000s, an outbreak of CL was observed after the Iraq War that spread beyond endemic populations and included foreign troops in the area [43,51]. The 1990s Afghani civil war experience also was notable for its outbreak of CL [52]. The war is estimated to have caused hundreds of thousands of CL cases in Afghanistan and among refugee populations in Pakistan [52,53].

## Old World CL and Other Conflict Zones in MENA

Knowledge of Old World CL in Syria and among its refugees is limited; however, we know even less about the situation in areas of Libya now controlled by the self-proclaimed Islamic State, or Daesh, and its allied extremist groups [54]. Both zoonotic and anthroponotic disease cycles have been identified in Libya; however, most of the published literature on CL in Libya focuses on the zoonotic form, which is caused by *L*. *major*. This form is responsible for the majority of CL cases in Libya, with *Ph*. *papatasi* as the main vector, and *Psymommys obesus* (fat sand rat) and *Meriones libycus* (Libyan jird) reported as disease reservoirs [55]. However, *L*. *tropica* anthroponotic CL has also been identified in Nalut and Bani Walid. Interestingly, both *L*. *infantum* and *L*. *donovani* have been identified in Nalut as causative for CL [55,56]. Outdoor activities like farming and construction work are highly correlated with disease emergence as a result of increasing exposure to sandfly bites [57,58]. Recently, the United Nations High Commissioner for Refugees (UNHCR) reported that 363,067 individuals have been displaced in Libya due to the ongoing unrest [59]. Re-emergence of CL from mass displacement could occur in areas that have had experience with CL, including Siret, Nalut, Garyan, Bani Walid, Kikla, and Ghudamis. Furthermore, about one million Libyan refugees have been displaced to Tunisia. Anecdotal reports from Tunisia, where refugee camps have been established [60], indicate that cases of leishmaniasis are on the rise, but there is minimal, if any, documentation.

Leishmaniasis is a hidden NTD in Yemen as well. Approximately 10,000 new cases are reported annually [61]. These cases are caused by both *L*. *tropica* and *L*. *infantum* in high altitude regions, including Sa’da, Amr’an, Al Bayda, Ibb, Al Dhale’a, Dhamar, and Sana’a [62,63]. Furthermore, *L*. *donovani*, *L*. *tropica*, and *L*. *infantum* cause CL in regions that belong to the Tihama Coastal Plain, such as Al Hudaydah, Hajjah, and Ta’izz [62]. The Regional Leishmaniasis Control Centre (RLCC) reports that half of the clinically resembling CL cases are mucocutaneous leishmaniasis and that Yemeni CL patients suffer from both shortage of CL treatment and inadequate response to treatment [64]. Moreover, the access to health care has been reduced significantly due to conflict in Yemen and absence of aid. As CL in Yemen is thought to be caused exclusively by an anthroponotic cycle, the disease prevalence will likely increase as the rubbish accumulation and lack of sewage system foster the perfect breeding sites for *Ph*. *sergenti* vector. Although no refugee camps have been deployed as a result of the current Yemeni conflict, many people are migrating to neighboring countries such as Saudi Arabia, which may lead to the spreading of anthroponotic CL in the southern Saudi regions. The situations in Libya and Yemen will need further monitoring.

## Discussion and Preliminary Recommendations

Areas of conflict provide for complex circumstances that make accurate data collection and humanitarian aid inaccessible and impractical. Additionally, there is ongoing dialogue about the efficacy of humanitarian aid in areas of conflict [65]. Especially with the loss of governmental control in many areas of Syria, Iraq, and parts of Libya to Daesh, policy recommendations are nearly impossible to implement in many regions of MENA [2].

Despite the difficulties of navigating current geopolitical circumstances in MENA, more can be done to address the CL situation in this region. Interventions to prevent and control the spread of CL must be multilateral in dimension and specific to local circumstances [66]. The following list of preliminary recommendations to prevent and control CL outbreaks highlights general policies that have already been proposed by organizations such as the WHO and the Centers for Disease Control [66–68], and emphasizes refugee camps and communities of displaced individuals living in regions of stability in MENA. The utmost priority of all interventions is to do no harm.

1. Continued improvement of living conditions and hygiene infrastructure for refugees. Clean water, food, and sanitation services aid basic survival while also aiding the prevention and control of CL among endemic refugee populations.
2. Implementation of mobile teams in refugee camps consisting of medical professionals experienced in diagnosing and treating CL. Responsibilities for mobile teams would include disease (and vector) detection, active surveillance, and providing health care treatment for patients with CL. Treatment includes sodium stibogluconate (Pentostam) and meglumine antimoniate (Glucantime), as well as alternatives therapies, such as cryotherapy, if there is a treatment shortage.
3. Collection of health impact assessments prior to the establishment of refugee camps in neighboring areas of conflict zones. For example, the extermination of animal reservoirs before settling displaced individuals can help avoid emerging outbreaks among refugee communities [7].
4. Implementation of services to address psychological and economic impacts of CL. The most devastating consequences of contracting CL are often socioeconomic and psychological.
5. Initiatives addressing community stigma surrounding skin lesions and papules associated with CL. Additional educational programs in refugee communities to raise awareness of CL also may be beneficial in preventing outbreaks.
6. Distribution of insecticide treatment, particularly in areas known to be endemic with anthroponotic cycle, to help prevent contagion. A recent Cochrane analysis has concluded that insecticides may be effective at reducing the incidence of CL; however, whether insecticides are best applied through indoor spraying, treatment of clothing and bed sheets, or use of nets remains undetermined [69].
7. Research and development to improve diagnosis, treatment, and prevention methods, as well as ongoing operational research, monitoring, and evaluation to confirm the effectiveness of existing approaches. All research and development initiatives should give due considerations to ethical issues of working with refugee populations.

The full extent of the Old World CL epidemic in Syria and in bordering countries, as well as in Libya and Yemen, remains mostly unknown. An adequate disease burden analysis depends on programs of active surveillance and disease detection, but these are few and far between due to the violence and instability. We may be witnessing an epidemic of historic and unprecedented proportions, but it has largely been hidden due to lack of specific information. The biggest limitation of this paper is the inability to access data due to the difficulties of gathering accurate and current information from regions of instability. Surveillance is even more challenging in the current refugee crises due to the unprecedented magnitude of population migration.

The most effective policies in addressing the potentially devastating CL situation that is emerging from some conflict zones in MENA are initiatives that will promote disease control while simultaneously promoting the survival of refugees. Provisions of clean water, food, hygiene services, and adequate shelter will improve the living conditions of refugees while simultaneously addressing many of the socioeconomic and environmental risk factors that make refugees highly susceptible to infectious diseases. For example, makeshift houses allow sandflies to come in close proximity to human beings and the lack of municipal services creates conditions that facilitate poor health outcomes.

Recommendations for research include the development of improved rapid diagnosis tests, possibly similar to the point-of-care diagnostic tests under development for VL [70]. Currently, diagnoses are performed by specialized dermatologists and can only be confirmed by a stained smear or culture from a skin lesion, which require laboratory settings. The lack of a rapid diagnosis test slows the process of diagnosis and leads to delayed treatment and greater risk for misdiagnosis of CL. Development of a commercially available vaccine for Old World CL should also be made a priority, as one does not currently exist even though it would enhance efficacy of disease and vector control programs [71,72]. A recent analysis confirms the cost-effectiveness for a vaccine that targets either New World CL [72] or Old World VL [73]. Additionally, research assessing how best to address the socioeconomic and psychological impacts of CL on patients as well as the cultural stigma of papules left by CL would facilitate a more well-rounded approach to confronting the consequences of CL outbreaks. These research projects should be specific to the dynamics of local communities and cultures. Micro-financing programs may alleviate some of the economic hardships often associated with CL; however, the feasibility of micro-financing programs in conflict-affected communities is still being debated [74].

A multifaceted, collaborative approach must be taken to control the incidence of CL [7,75], with priority given to initiatives that will not only aid in the prevention and control of CL but also improve the living conditions and survival of refugee populations. The World Health Assembly already adopted a resolution in 2007 to address the global burden of leishmaniasis [67], but immediate action must be taken to address the spreading burden of CL in the Middle East. By no fault of their own, refugees and displaced individuals are often fleeing from one unimaginable circumstance of horror and violence to another of poverty and disease. International communities have a responsibility to pay greater attention to this pressing issue, and it is imperative that proactive measures are taken to establish efficient and sustainable initiatives aimed at diagnosing, treating, and preventing CL as the conflicts in Syria, Iraq, Libya, and Yemen continue.
